# Flow cytometric analysis of the CD4+ TCR Vβ repertoire in the peripheral blood of children with type 1 diabetes mellitus, systemic lupus erythematosus and age-matched healthy controls

**DOI:** 10.1186/1471-2172-14-33

**Published:** 2013-08-03

**Authors:** Flora Tzifi, Maria Kanariou, Marianna Tzanoudaki, Constantinos Mihas, Evangelia Paschali, George Chrousos, Christina Kanaka-Gantenbein

**Affiliations:** 1 First Department of Pediatrics, Division of Endocrinology, Diabetes and Metabolism, National Kapodistrian University of Athens, “Aghia Sophia” Children’s Hospital, Thivon & Papadiamantopoulou street, 11527 Athens, Greece; 2 Department of Immunology & Histocompatibility, Specific Center & Referral Center for Primary Immunodeficiencies - Paediatric Immunology, “Aghia Sophia” Children’s Hospital, Athens, Greece; 3 Internal Medicine Department, Kimi General Hospital “G. Papanicolaou”, Kimi, Greece

**Keywords:** TCR Vβ repertoire, Flow cytometry, T1DM, SLE, Children

## Abstract

**Background:**

Data regarding the quantitative expression of TCR Vβ subpopulations in children with autoimmune diseases provided interesting and sometimes conflicting results. The aim of the present study was to assess by comparative flow cytometric analysis the peripheral blood CD4+ TCR Vβ repertoire of children with an organ-specific autoimmune disorder, such as type 1 diabetes mellitus (T1DM), in comparison to children with a systemic autoimmune disease, such as Systemic Lupus Erythematosus (SLE) in comparison to healthy age-matched controls of the same ethnic origin. The CD4+ TCR Vβ repertoire was analysed by flow cytometry in three groups of participants: a) fifteen newly diagnosed children with T1DM (mean age: 9.2 ± 4.78 years old), b) nine newly diagnosed children with SLE, positive for ANA and anti-dsDNA, prior to treatment (mean age: 12.8 ±1.76 years old) and c) 31 healthy age-matched controls (mean age: 6.58 ± 3.65 years old), all of Hellenic origin.

**Results:**

CD4 + TCR Vβ abnormalities (± 3SD of controls) were observed mainly in SLE patients. Statistical analysis revealed that the CD4 + Vβ4 chain was significantly increased in patients with T1DM (p < 0.001), whereas CD4 + Vβ16 one was significantly increased in SLE patients (p < 0.001) compared to controls.

**Conclusions:**

CD4 + Vβ4 and CD4 + Vβ16 chains could be possibly involved in the cascade of events precipitating the pathogenesis of T1DM and SLE in children, respectively.

## Background

Autoimmunity involves complex pathophysiological processes; however, no comprehensive model of inflammatory induction has been unraveled as yet. Genetic and environmental immunologic factors, several hormones and stress have been implicated in the pathogenesis of autoimmune disorders [1-4]. Moreover, the T cell receptor (TCR), with its extended repertoire derived from somatic recombination mechanisms, plays a potential role in human autoimmune disease [5]. Animal and human genetic studies have shown that skewing of TCR Vβ repertoire is a characteristic feature of some autoimmune disorders although still controversy exists on that issue [6,7].

TCR Vβ repertoire is assessed with two methodologies, CDR3 spectratyping, which is a genetic assay that provides mainly qualitative information about TCR Vβ clonality [8,9], and flow cytometry analysis, which provides a quantitative assessment of TCR Vβ clones and is well established in the clinical setting [10,11]. In T cell-mediated organ-specific autoimmune diseases, such as type 1 diabetes (T1DM), TCR Vβ repertoire analysis, using both spectratyping and flow cytometry, produced conflicting findings [12,13]. In systemic autoimmune diseases, like Systemic Lupus Erythematosus (SLE), TCR Vβ analysis has been conducted in adult populations using the spectratyping method and a marked oligoclonality of the TCR Vβ repertoire has been reported, which is more prominent in patients with active disease [14,15]. It is likely that children with SLE display a similar phenotype, although no data exist regarding the expression of the Vβ chains in the pediatric population. The marked skewing of the TCR Vβ repertoire observed in some SLE patients has not been found in T1DM patients, although a comparative study in a pediatric population between an organ-specific disease, such as T1DM, and a systemic autoimmune one, such as SLE, to our knowledge, has not been conducted as yet.

The aim of this study was to compare the peripheral blood CD4+ TCR Vβ repertoire in children with T1DM and SLE, in comparison to healthy age-matched controls of the same ethnic origin with the use of the flow cytometry assay.

## Results

### a) Healthy children

Complete blood count values, immunoglobulin levels and lymphocyte subpopulation counts were normal in all healthy children. Autoantibodies, either SLE-specific or diabetes speficic (ANA, anti-dsDNA, ICA), were evaluated and they were negative in all controls tested. There was an unavoidable male preponderance in the tested children (boys: 80.6%), due to the higher frequency of anatomical abnormalities needing surgical reconstruction in boys.

Mean values and standard deviations (SD) of each distinct Vβ family percentage on CD4+ lymphocytes of the 31 studied children are shown in Table 1. TCR Vβ usage appears to be non-random varying from low values of 0.0% for Vβ4 and Vβ7.2 to high values of 12.8% for Vβ2. Additionally, in one individual the value of the Vβ7.2 chain was 0.0%. The known polymorphism (null expression) in Vβ20 subfamily was not detected in the population tested.

**Table 1:** CD4 + Vβ repertoire usage in healthy children

**Vb subfamily**	**Mean percentage**	**min (%)**	**max(%)**	**SD**
**Vb1**	3.21	1.8	4.9	.66
**Vb2**	7.86	4	12.8	2.33
**Vb3**	4.23	1	8.5	2.22
**Vb4**	.76	.0	2.3	.58
**Vb5.1**	4.43	1.8	6.9	1.48
**Vb5.2**	1.39	.5	4.8	.97
**Vb5.3**	1.69	.6	3.5	.73
**Vb7.1**	1.85	.4	4.1	.89
**Vb7.2**	1.71	.0	4.9	1.07
**Vb8**	3.95	2	6.4	1.02
**Vb9**	2.90	.8	12.6	2.13
**Vb11**	1.49	.4	2.9	.70
**Vb12**	4.44	1.6	9.7	1.65
**Vb13.1**	4.23	2.1	7.4	1.35
**Vb13.2**	2.58	.2	6.8	1.42
**Vb13.6**	1.86	.8	3.5	.61
**Vb14**	4.00	1.4	9.6	1.78
**Vb16**	2.03	.6	5.1	.99
**Vb17**	3.88	.1	5.4	1.33
**Vb18**	1.34	.2	3.9	.90
**Vb20**	4.08	1.2	7.6	1.75
**Vb21.3**	2.47	.7	3.6	.71
**Vb22**	2.84	.7	7.5	1.26
**Vb23**	1.17	.2	4	.90

TOTAL: 70.39 (CD4+).

CD4 + TCR Vβ repertoire usage in 31 healthy Greek Children. Minimum (min) and maximum (max) values obtained, as well as the mean percentages of the 24 CD4+ TCR Vβ chains are displayed. *SD* standard deviation.

**Figure 1:**
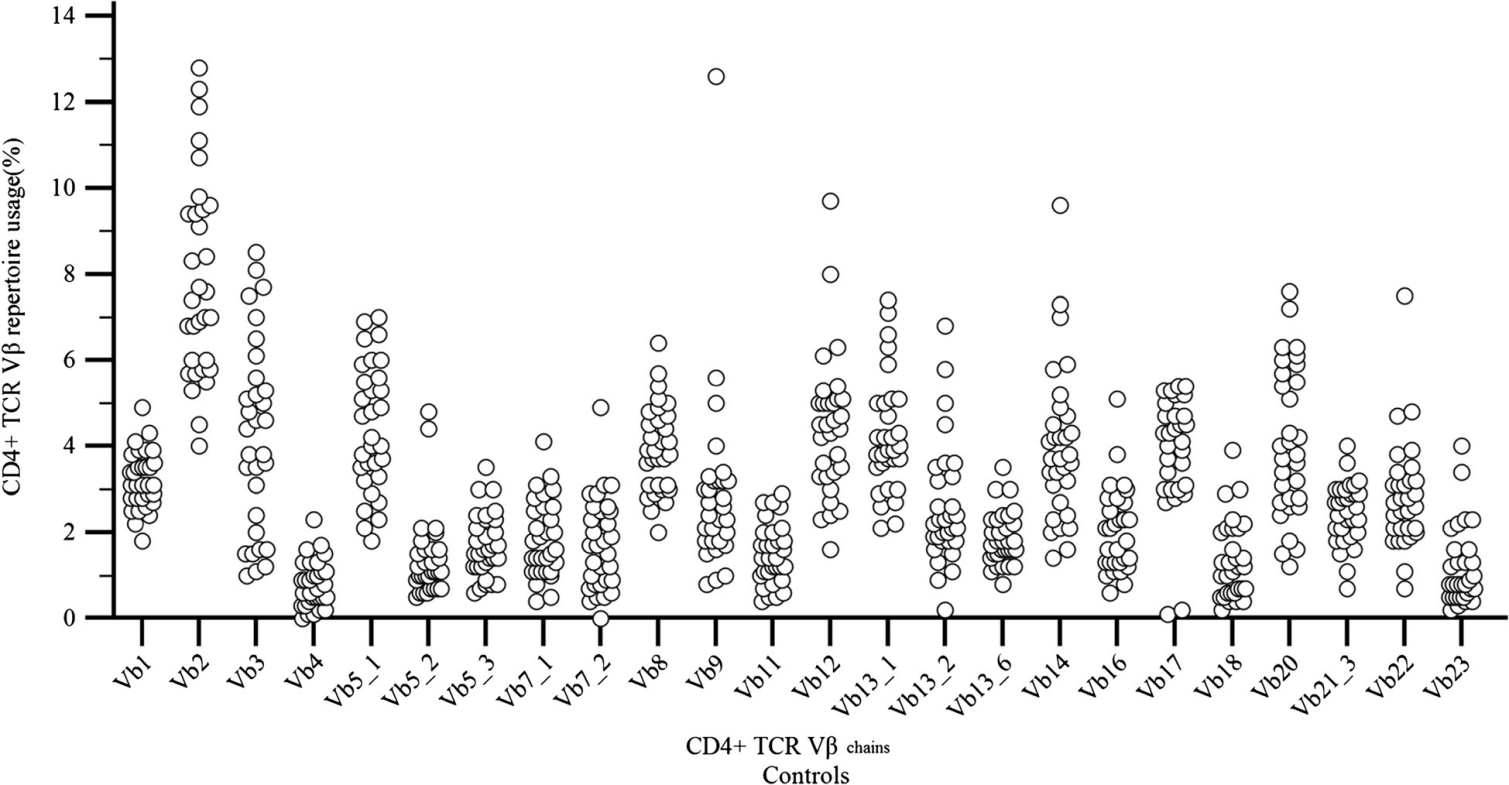
***CD4 + TCR Vβ chains quantitative expression in healthy children*.** CD4 + TCR Vβ repertoire usage (values %) in healthy controls revealed that increased usage of Vβ subpopulations can be observed sporadically even in healthy children.

The increased/decreased usage of a Vβ subfamily has arbitrarily been defined as mean value ± 2 or 3 standard deviations (mean value is the one derived from the control sample of the present study) [10,11]. Of the children tested, 16 individuals had increased usage (expressed as mean value + 2 or 3SD) of one or more Vβ lymphocyte populations and these findings concerned different Vβ chains in each subject. Seven children had an increased usage of mean value + 2SD and nine an increased usage of mean value + 3SD. Of these 16 subjects, 11 controls presented with an increased usage in only one chain (Vβ1, Vβ3, Vβ5.1, Vβ5.2, Vβ5.3, Vβ7.1, Vβ12, Vβ14, Vβ16, Vβ20, Vβ22), 4 controls had increased usage in two Vβ subpopulations (Vβ4-Vβ2, Vβ5.1-Vβ21.3, Vβ13.1-Vβ13.6, Vβ7.2-Vβ13.2 ) and one control in five ones (Vβ5.2, Vβ7.4, Vβ8, Vβ9, Vβ18) (Figure 1). No discrepancies were noticed in the Vβ11 and Vβ17 chains.

Enrolled infants and children were separated into three age groups, which were compared in order to find out possible differences in Vβ expression at different age groups. Table 2 displays median values and interquartile range of the CD4+ TCR Vβ repertoire in the aforementioned subgroups as well as in the total population of the study. Statistical analysis revealed no significant difference for each of the 24 TCR Vβ subfamilies in the CD4+ T lymphocytes.

**Table 2:** TCR Vβ repertoire usage by age groups

**Vβ repertoire usage**	**Age categories**											
	**18 months-4 years**			**5-9 years**			**10-14 years**			**Total**		
	**Median**	IQR*	**p(18 m-4/5-9)†**	**Median**	IQR*	**p(5-9/10-14)**	**Median**	IQR*	**p(18 m-4/10-14)**	**Median**	IQR*	**p(overall)**
Vβ3	4.70	3.10	.080	3.35	2.90	.121	5.20	2.60	.831	4.40	3.60	.156
Vβ5.3	1.50	.70	.692	1.35	.90	.033	2.30	.90	.059	1.60	1.10	.068
Vβ7.1	1.50	.85	.643	1.25	.90	.022	2.60	1.20	.050	1.60	1.50	.048
Vβ16	1.70	1.10	.972	2.10	1.00	.248	2.50	1.40	.319	1.95	1.40	.472
Vβ9	2.35	1.30	.915	2.70	1.60	.825	2.60	1.40	.943	2.50	1.40	.984
Vβ17	4.05	1.85	.695	3.90	1.70	.690	4.30	1.80	.337	4.20	1.70	.652
Vβ20	3.35	1.70	.339	4.10	3.10	.838	5.10	2.30	.145	3.80	3.00	.346
Vβ18	.85	.90	.427	1.15	.80	.093	2.10	1.20	.027	1.20	1.50	.063
Vβ5.1	3.85	1.65	.448	4.85	3.00	.935	4.00	2.80	.670	4.20	2.30	.767
Vβ8	4.00	1.30	.741	4.00	1.80	.236	3.70	1.40	.374	3.90	1.60	.472
Vβ13.1	3.90	1.30	.692	3.75	2.10	.595	4.20	1.10	.859	3.90	1.50	.856
Vβ13.6	1.55	.60	.128	1.85	.70	.594	2.10	.50	.058	1.80	.80	.116
Vβ12	4.70	1.65	.717	4.50	2.10	.462	4.20	2.50	.125	4.50	1.80	.367
Vβ5.2	1.05	.90	.643	1.10	.50	.712	1.20	.90	.617	1.10	.90	.824
Vβ2	7.90	3.25	.448	6.95	3.80	.513	7.60	3.10	.722	7.40	3.70	.688
Vβ21.3	2.35	.95	.222	2.65	1.00	.967	2.80	.70	.319	2.60	1.00	.418
Vβ23	.80	.65	.740	.75	1.10	.189	1.30	1.50	.074	.80	1.10	.188
Vβ1	3.10	.55	.425	3.25	.70	.594	3.50	1.10	.198	3.10	.80	.414
Vβ14	4.20	1.75	.306	3.50	1.90	.967	3.70	1.50	.226	3.70	2.00	.418
Vβ11	1.20	.95	.921	1.50	.90	.190	1.80	1.40	.212	1.40	1.00	.342
Vβ22	2.70	1.20	.766	2.55	1.10	.165	3.10	1.40	.269	2.70	1.10	.348
Vβ7.2	1.35	1.70	.113	2.25	1.90	.487	1.70	1.10	.355	1.70	1.70	.261
Vβ13.2	1.85	.95	.129	2.80	1.80	1.000	2.40	.30	.042	2.20	1.50	.110
Vβ4	.85	.65	.741	.70	.70	.411	.50	.90	.238	.70	.80	.484

No age-related differences in CD4 + Vβ expression were found among the three age groups of healthy Greek children studied.

* *IQR* Interquartile range (75^th^ - 25^th^ percentile).

† The numbers in brackets indicate the two age groups that were used for the comparison with the corresponding p-value.

### b) T1DM children

Assessment of the CD4+ TCR Vβ repertoire was performed in two ways: a) Vβ repertoire of each T1DM patient was compared with the Vβ mean values of the control group and b) statistical analysis was performed, in order to detect the presence of CD4 + Vβ lymphocyte clones in the group of T1DM patients with different quantitative expression in comparison to the control group.

**Figure 2:**
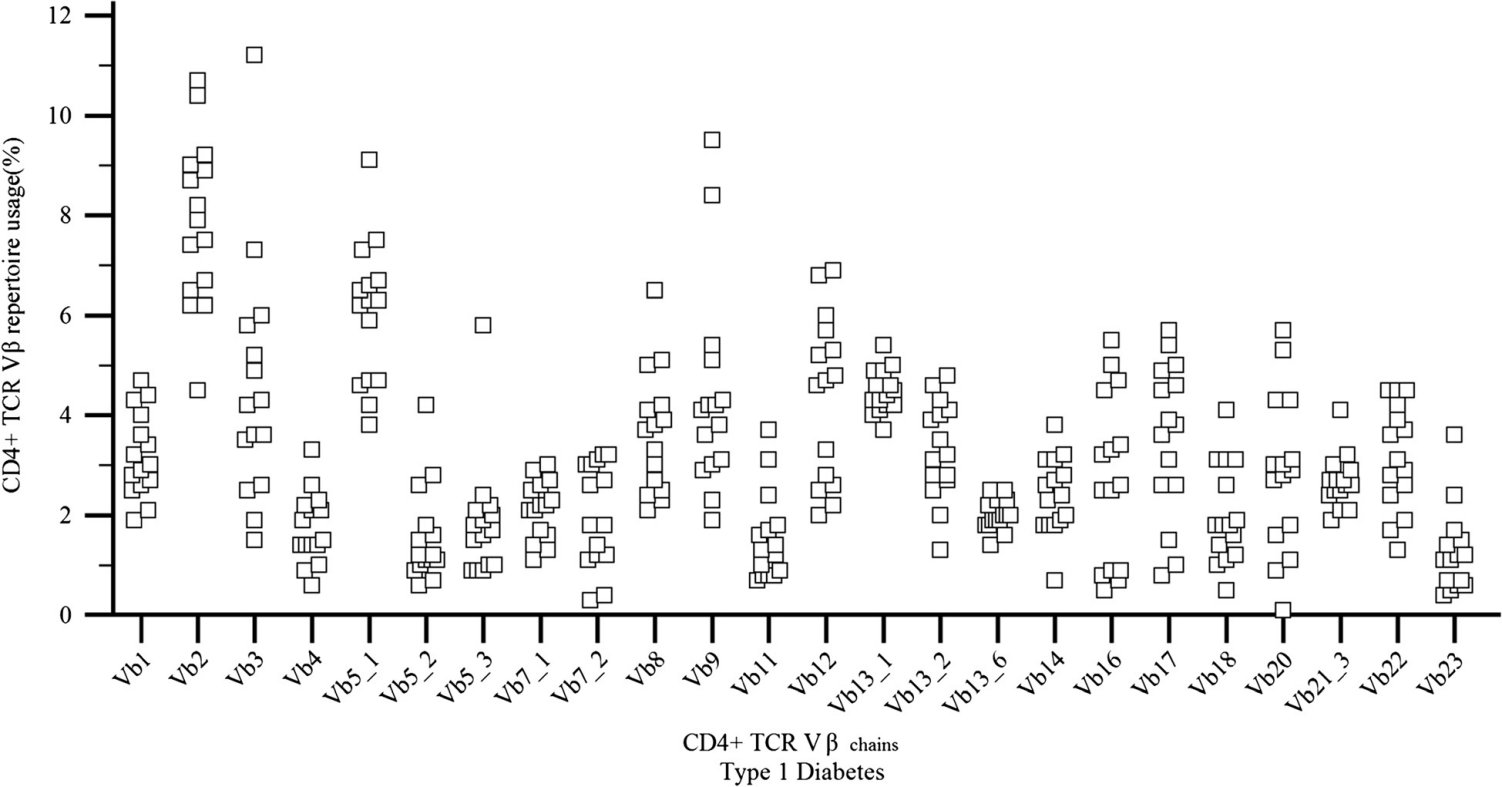
***CD4 + TCR Vβ values (%) in T1DM children.*** CD4 + TCR Vβ repertoire usage (values %) in T1DM children resembles the one observed in healthy children with the exception of Vβ4 subpopulation.

**Figure 3:**
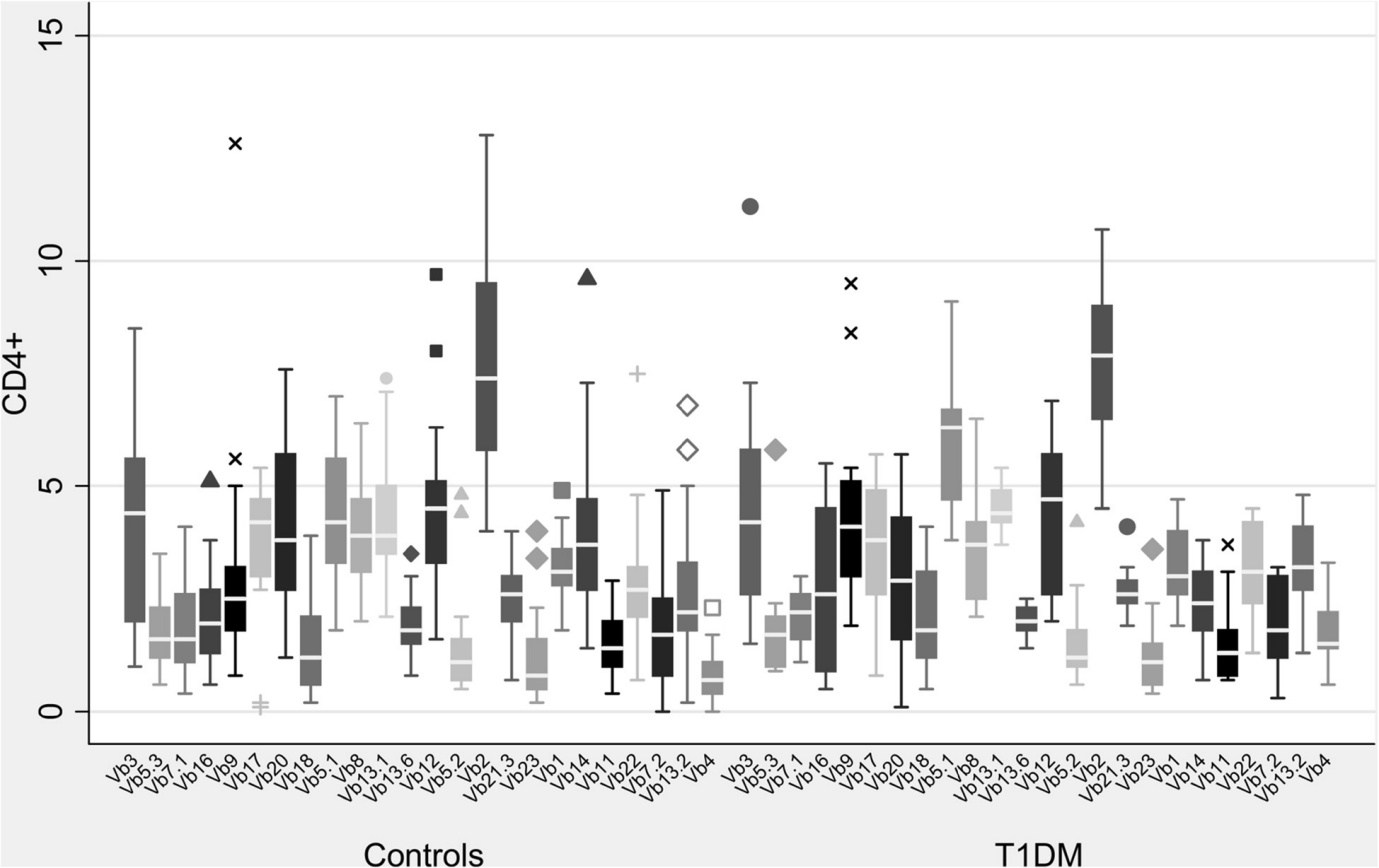
***Comparison of CD4+ Vβ repertoire between T1DM and healthy children.*** Median values of the CD4+ TCR Vβ repertoire in healthy children and in children with T1DM are presented. The CD4 + Vβ4 chain presented with a significantly increased expression (p < 0.001) when compared to healthy age-matched individuals.

**Figure 4:**
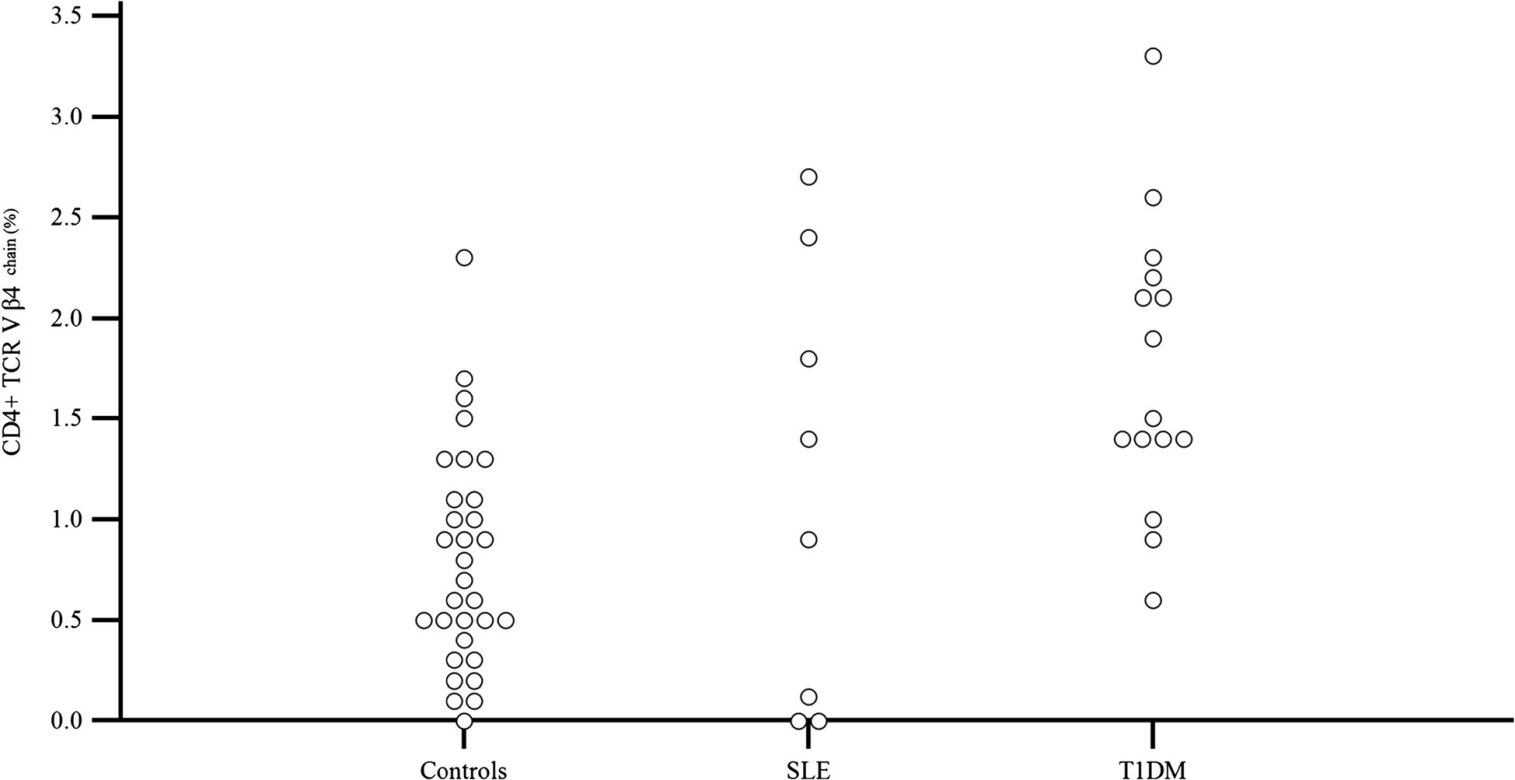
***Comparison of CD4 + Vβ4 chain values among the 3 groups of children studied*.** Values (%) of the CD4 + Vβ4 chain are higher in the majority of T1DM children in comparison to controls.

The increased/decreased usage of a Vβ subfamily was defined as control mean value ± 2 or 3 standard deviations [10,11]. Five patients (33%) presented no abnormalities in all CD4 + Vβ chains values when compared to controls (Table 3, Figure 2). One T1DM patient had decreased usage of Vβ14 (0.7%, mean value: 4.00 ± 1.78) and in another one Vβ20 value was 0.1% (mean value: 4.08 ± 1.75). None of the healthy individuals had such low usage of these Vβ chains. Increased usage of several CD4 + Vβ chains (Vβ3, Vβ4, Vβ5.1, Vβ5.3, Vβ8, Vβ11, Vβ18, Vβ16 and Vβ21.3) was identified sporadically in T1DM patients. With the exception of Vβ11 chain, increased usage of the mentioned Vβ subpopulations were also found in healthy individuals. Although the CD4 + Vβ4 lymphocyte population increased usage was found in 5 T1DM patients when compared to the mean value of controls, statistical analysis showed a significant difference between the two groups (p < 0.001) concerning the CD4 + Vβ4 subfamily (Table 4, Figure 3). This is expected, since values of the controls ranged between 0.0% and 2.1% while T1DM values ranged from 0.6% to 3.3%. It should be mentioned that 17 healthy children had values of the CD4 + Vβ4 chain < 1% and 12 T1DM children had values > 1% (Figure 4). Taking into account the very low number of CD4 + Vβ4 cells analyzed and that the maximum coefficient of variation (CV) was 20%, statistical analysis was adjusted to this CV. As shown in Table 5, the differences remained significant even after “narrowing” the mean values, adjusting for a 20% difference in the mean values of our measurements.

**Table 3:** CD4 + Vβ chains usage in T1DM children

**T1DM children**	**CD4+ TCR Vβ Increased usage**	**CD4 + TCR Vβ Decreased usage**
Patient 1	Vβ5.1 (7.3%, Μ + 2SD)	None
Patient 2	Vβ5.3 (5.8%, Μ + 3SD)	None
Patient 3	Vβ16 (5%, Μ + 3SD)	None
	Vβ5.1 (7.5%, Μ + 2SD)	
	Vβ8 (6.5%, Μ + 2SD)	
	Vβ21.3 (4.1%, Μ + 2SD)	
Patient 4	None	None
Patient 5	Vβ16 (5.5%, Μ + 3SD)	Vβ14 (0.7%, Μ-2SD)
	Vβ18 (4.1%, Μ + 3SD)	
	Vβ11 (3.1%, Μ + 2SD)	
Patient 6	None	None
Patient 7	None	None
Patient 8	Vβ3 (11.2%, Μ + 3SD)	None
Patient 9	Vβ4 (2.6%, Μ + 3SD)	None
Patient 10	None	None
Patient 11	Vβ4 (2.3%, Μ + 2SD)	None
Patient 12	Vβ4 (2.1%, Μ + 2SD)	None
Patient 13	None	None
Patient 14	Vβ4 (2.2%, Μ + 2SD)	None
Patient 15	Vβ5.1 (9.1%, Μ + 3SD)	Vβ20 (0.5%, Μ-2SD)
	Vβ4(3.3%, Μ + 3SD)	

CD4 + TCR Vβ repertoire observed in T1DM patients in comparison to controls was similar. Low usage of the Vβ chains is rarely found in patients with T1DM.

**Table 4:** Results of statistical analysis between T1DM children and controls and SLE children and controls

	**Group**							
	**Controls**		**SLE**			**T1DM**		
	**Median**	**iqr**	**Median**	**iqr**	**p**	**Median**	**iqr**	**p**
Vb3	4.40	3.60	5.60	2.40	.200	4.20	3.20	.833
Vb5.3	1.60	1.10	1.40	1.00	.661	1.70	1.10	.851
Vb7.1	1.60	1.50	1.70	1.40	.475	2.20	1.00	.181
**Vb16**	1.95	1.40	5.62	4.30	**<.001**	2.60	3.60	.252
Vb9	2.50	1.40	3.60	2.60	.019	4.10	2.10	.002
Vb17	4.20	1.70	2.40	3.26	.029	3.80	2.30	.515
Vb20	3.80	3.00	3.85	2.75	.543	2.90	2.70	.044
Vb18	1.20	1.50	1.00	1.00	.464	1.80	1.90	.027
Vb5.1	4.20	2.30	4.00	2.40	.498	6.30	2.00	.003
Vb8	3.90	1.60	2.80	2.90	.175	3.70	1.70	.302
Vb13.1	3.90	1.50	5.10	1.10	.067	4.40	.70	.119
Vb13.6	1.80	.80	1.80	1.40	.791	2.00	.50	.145
Vb12	4.50	1.80	6.30	4.64	.013	4.70	3.10	.879
Vb5.2	1.10	.90	1.70	1.20	.650	1.20	.80	.438
Vb2	7.40	3.70	7.00	4.67	.318	7.90	2.50	.815
Vb21.3	2.60	1.00	3.48	.80	.006	2.60	.50	.605
Vb23	.80	1.10	.95	.51	.986	1.10	.90	.557
Vb1	3.10	.80	3.45	2.05	.564	3.00	1.40	.869
Vb14	3.70	2.00	3.35	3.09	.835	2.40	1.30	.001
Vb11	1.40	1.00	1.79	1.40	.169	1.30	1.00	.842
Vb22	2.70	1.10	2.10	2.22	.085	3.10	1.80	.236
Vb7.2	1.70	1.70	3.15	1.45	.010	1.80	1.80	.241
Vb13.2	2.20	1.50	3.25	1.35	.012	3.20	1.40	.017
**Vb4**	.70	.70	1.15	2.04	.542	1.50	.80	**<.001**

Statistical analysis of the CD4+ TCR Vβ lymphocytes between a) SLE patients and controls, b) T1DM patients and controls. Only the Vβ16 chain presented with significantly increased usage in SLE children and the Vβ4 chain in T1DM ones.

**Table 5:** Adjusted statistical analysis in CD4 + Vβ4 chain

**Vβ4**	**Group**	**Mean**	**Std. Err.**	**Std. Dev.**	**[95% Conf.**	**Interval]**			
	Controls	0.797	0.097	0.541	0.598	0.995	<.001		
	T1DM	1.740	0.183	0.710	1.347	2.133			
**Vβ4**	**Group**	**“New” mean**			**Std. Err.**	**Std. Dev.**	**[95% Conf.**	**Interval]**	**p**
	Controls	0.956	+20% of the original value		0.097	0.541	0.598	0.995	0.026
	T1DM	1.392	-20% of the original value		0.183	0.710	1.347	2.133	

Adjustment of statistical analysis for the CD4 + Vβ cells for a maximum CV of 20% showed that differences between healthy controls and T1DM children remained significant. CV: coefficient of variation.

### c) SLE children

**Figure 5:**
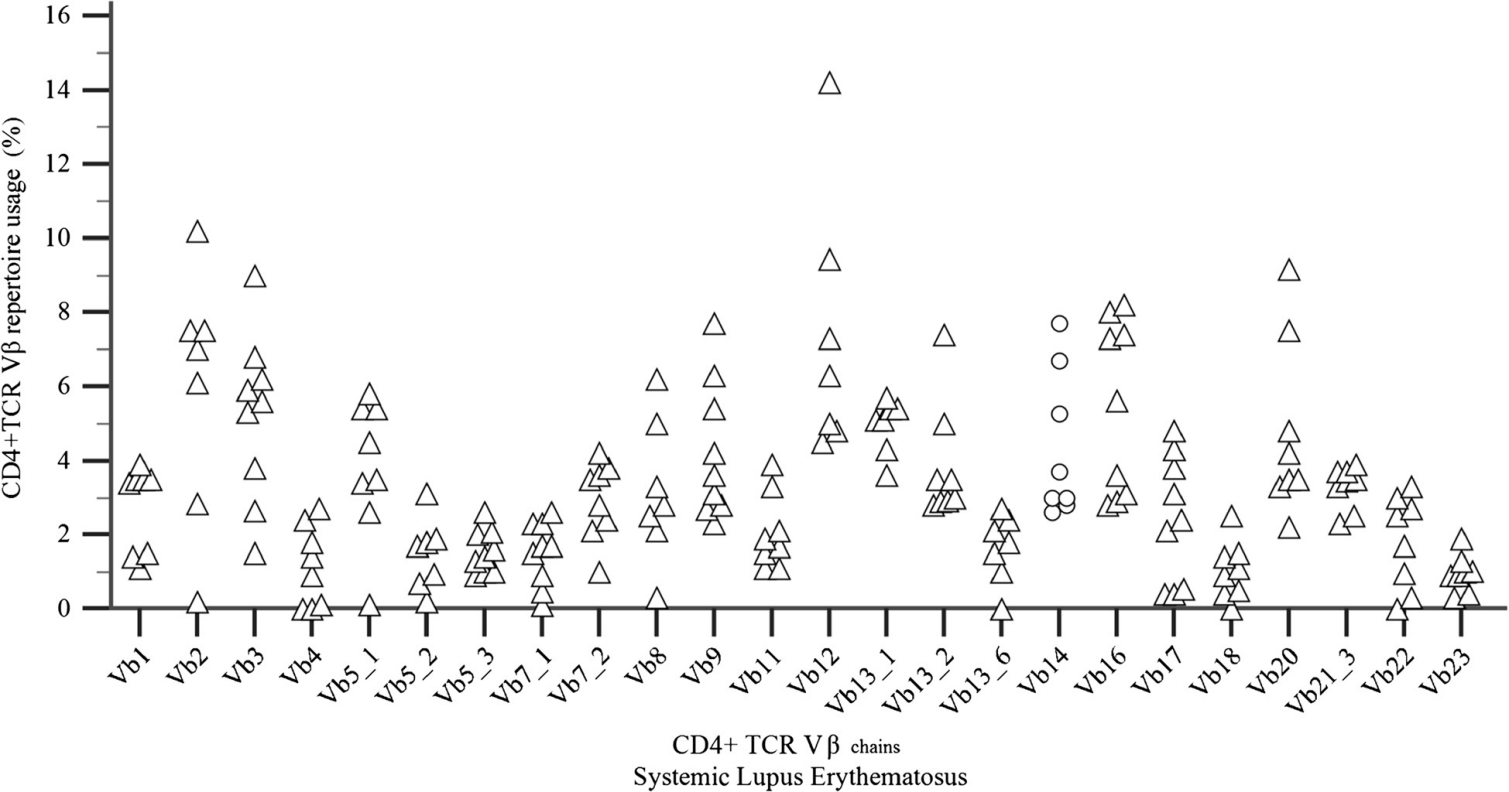
***CD4 + TCR Vβ repertoire usage in SLE children.*** CD4 + TCR Vβ repertoire usage (values %) in SLE children is quite different to the one of healthy children and T1DM patients. Many SLE children had very low usage of several Vβ chains.

**Figure 6:**
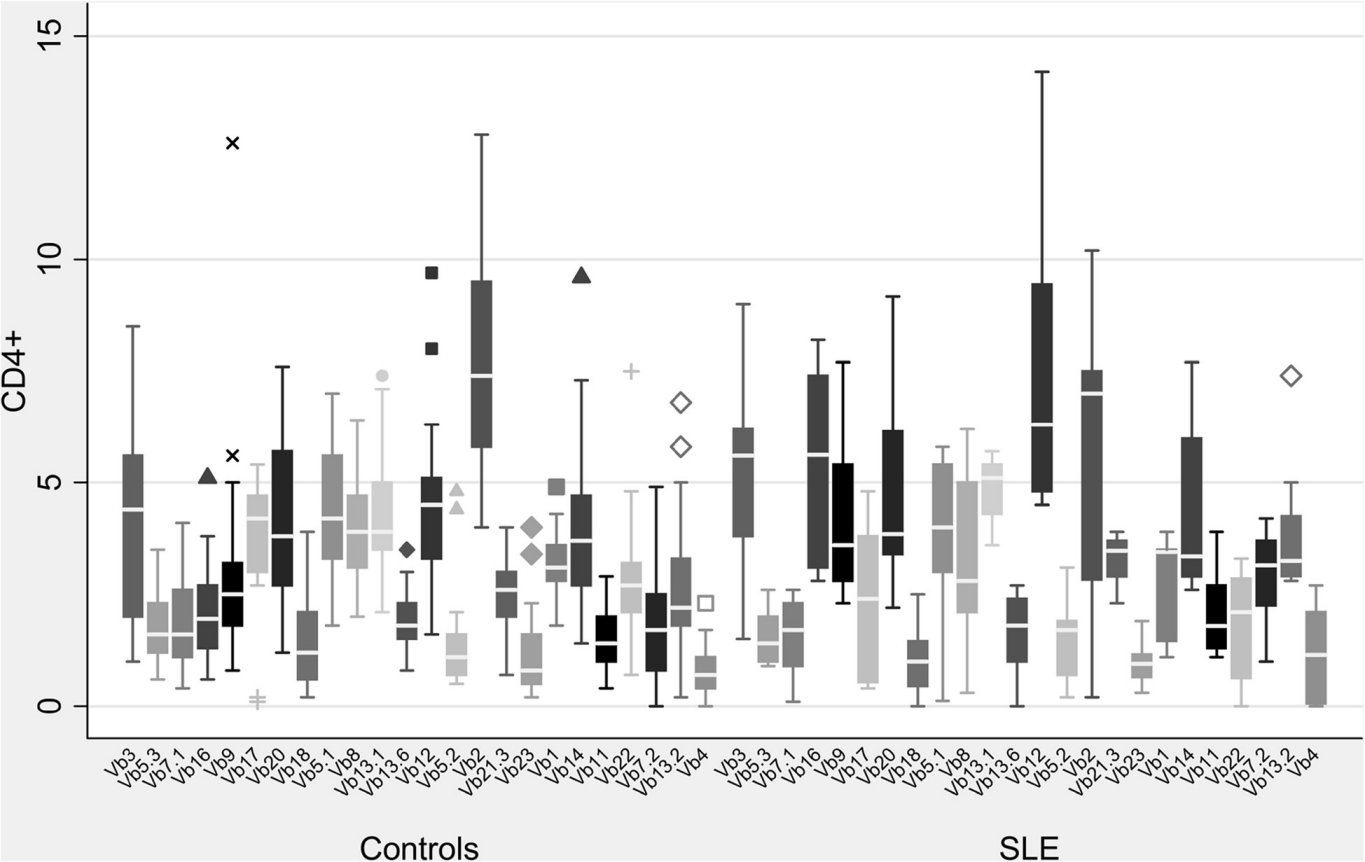
***Comparison of CD4+ Vβ repertoire between SLE and healthy children.*** Median values of the CD4+ TCR Vβ repertoire in healthy children and in children with SLE are presented. The CD4 + Vβ16 chain presented with a significantly increased expression (p < 0.001) when compared to healthy age-matched individuals.

**Figure 7:**
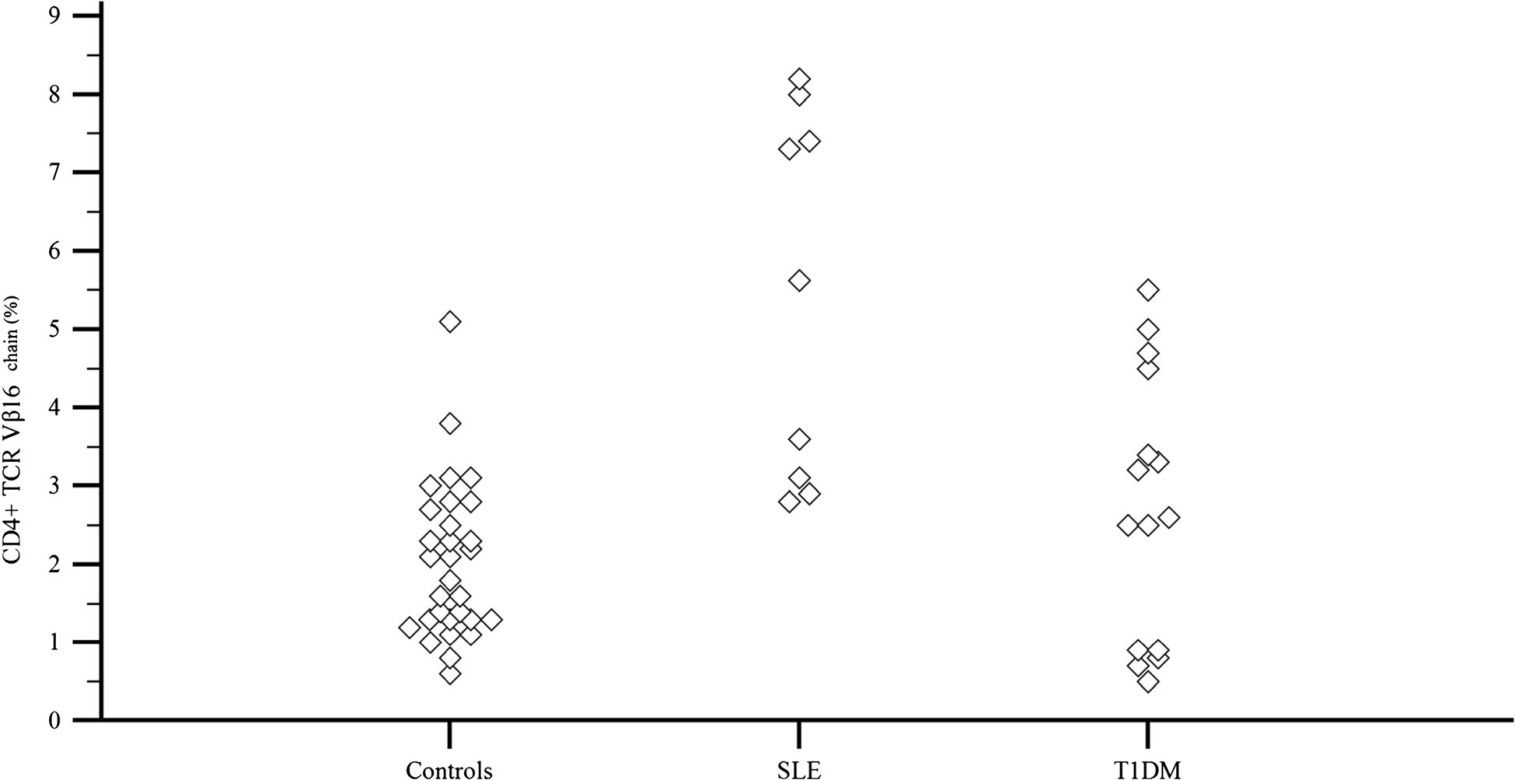
***Comparison of CD4 + Vβ16 chain values among the 3 groups of children studied*.** Values (%) of the CD4 + Vβ16 chain are higher in the majority of SLE children in comparison to controls.

In the SLE group, skewing in CD4 + Vβ subpopulations was detected in the majority of patients when compared to healthy individuals. In contrast to healthy children and T1DM patients, which presented with a similar CD4 + TCR Vβ repertoire pattern, 7 of the 9 SLE children tested displayed concurrently marked increased (mean value ± 3SD) and decreased usage (with even values of 0.0%) of several CD4 + Vβ subpopulations. This was noteworthy in one SLE patient who displayed increased usage of the CD4+ Vβ12 (9.44%), Vβ16 (5.6%) and Vβ20 (9.17%) and a decreased one of Vβ2 (2.83%) and Vβ5.1 (0.12%) chains and in another one patient with high values of CD4+ Vβ3 (9%), Vβ12 (14.2%) and Vβ16 (7.3%) chains and very low values of CD4+ Vβ2 (0.2%), Vβ5.2 (0.2%), Vβ7.1 (0.1%) and Vβ22 (0.3%) (Table 6, Figure 5). Markedly low values were found in the following CD4 + Vβ chains: Vβ2 (in 2 patients), Vβ22 (in 2 patients), Vβ5.1, Vβ5.2, Vβ7.1, Vβ8, Vβ13.6, and Vβ18. The CD4 + Vβ4 subpopulation had values of 0.0% in three SLE patients. However, this finding, as previously mentioned, was noticed in healthy children as well. Increased usage of Vβ16 was found in 5, of Vβ4 in 2, of Vβ20 in 2 and of Vβ12 in 2 patients. It should be mentioned, though, that there were two SLE patients with a normal repertoire at initial diagnosis. When statistical analysis was performed in order to compare the CD4 + Vβ expression between SLE and healthy children, significant differences in TCR Vβ quantitative expression were noticed only for the Vβ16 chain (p <0.001) (Table 3, Figure 6). This was expected, since five SLE patients presented with higher values of the CD4 + Vβ16 chain than those of the control group (mean value + 3SD) (Figure 7). Only one healthy individual presented an increased usage of CD4 + Vβ16, but he displayed no other discrepancies of the rest CD4 + Vβ subfamilies.

**Table 6:** Skewing of the CD4 + Vβ repertoire in SLE children

**Patients with ANA & anti-dsDNA**	**CD4+ TCR Vβ increased usage**	**CD4 + TCR Vβ decreased usage**
Patient 1	None	None
Patient 2	Vβ16 (7.4%, Μ + 3SD)	None
Patient 3	Vβ4 (2.4%, Μ + 3SD)	Vβ18 (0%, Μ-2SD)
Patient 4	Vβ16 (8.2%, Μ + 3SD)	Vβ8 (0.3%, Μ-3SD)
		Vβ13.6 (0%, Μ-3SD)
Patient 5	Vβ13.2 (7.4%, Μ + 3SD)	
	Vβ4 (2.7%, Μ + 3SD)	
Patient 6	Vβ16 (5.6%, Μ + 3SD)	Vβ5.1 (0.12%, Μ-2SD) Vβ2 (2.83%, Μ-2SD)
	Vβ20 (9.17%, Μ + 3SD)	
	Vβ12 (9.44%, Μ + 3SD)	
Patient 7	None	None
Patient 8	Vβ16 (8%, Μ + 3SD)	Vβ22 (0%, Μ-2SD)
	Vβ20 (7.5%, Μ + 2SD)	
Patient 9	Vβ3 (9%, Μ + 2SD)	Vβ7.1 (0.1%, Μ-2SD) Vβ5.2 (0.2%, Μ-2SD) Vβ2 (0.2%, Μ-2SD) Vβ22 (0.3%, Μ-2SD)
	Vβ16 (7.3%, Μ + 3SD)	
	Vβ12 (14.2%, Μ + 3SD)	

Discrepancies of the CD4+ TCR Vβ repertoire observed in SLE patients at initial diagnosis. *M* mean value (derived from controls sample), *SD* standard deviation.

### d) Findings of T lymphocyte populations by flow cytometric analysis

The basic immunophenotyping findings of the T1DM and SLE groups, when compared to controls, were as follows: a) Increased expression of CD69 molecule in two SLE patients and in one T1DM patient, b) elevated percentage of the CD3+ TCRγδ lymphocyte population (20%) in two SLE patients, c) increased expression of the HLA-DR + cells in 2 of the nine SLE children tested and d) absolute number of B lymphocytes (CD19+) beyond normal values in one SLE and in one T1DM patient. No difference in the CD5 + CD19+ expression was noticed among the three groups and the values (%) of the CD5 + CD19+ populations ranged between 0.1 and 1.2% in all individuals tested.

## Discussion

Flow cytometric analysis of the CD4 + TCR Vβ repertoire performed in three different groups of children of Greek origin, healthy, T1DM and SLE ones, provided results similar to previous published studies, but with some differences.

As far as healthy children are concerned, the results of this study support the non-random usage of TCR Vβ subfamilies in Greek children, as observed in previous studies on normal adults and children [11,16,17]. There is also no statistical difference in T cell repertoire among the three pediatric age groups studied and this is in accordance to another pediatric population studied in the United Kingdom [11]. Many healthy individuals had also sporadically increased expression of CD4 + Vβ chains as previously shown [11,16,17]. To our knowledge, analysis of the CD4 + Vβ repertoire expression pattern in Greek adults has not been conducted so far, so comparison with values of adults of the same ethnic origin could not be performed.

T1DM children had an almost same CD4 + Vβ repertoire pattern with healthy individuals, with the exception of the CD4 + Vβ4 chain which had increased expression in T1DM patients when compared to controls. Previous studies have shown similar results, that is either no differences between the two groups [12], or increased usage of other chains like the Vβ7 one [13,18]. Some putative explanations for the absence of consistency in the results among several studies could be the scarcity of published studies in human populations, the small size of the samples tested both in our study and others, as well as the different methodologies applied for the TCR repertoire assessment. It is still unclear though, whether the increased expression of CD4 + Vβ4 in newly diagnosed T1DM children of Hellenic origin is a constant finding in later stages of diabetes or not. Large-scale multicenter longitudinal studies are required to clarify this issue. A similar confirmed polymorphism of the TCRV20S1 gene was found in the healthy Sardinian population, but further research failed to relate this polymorphism with the high incidence of T1DM in Sardinians [19].

In contrast to T1DM patients, in SLE patients skewing of the CD4 + Vβ repertoire is far more prominent. This finding is in accordance to previous studies published in adults [14,15]. CDR3 spectratyping performed in the peripheral blood of 20 adult SLE patients has shown a prominent usage of the Vβ16 chain among other Vβ subpopulations (Vβ2, Vβ8, Vβ11, Vβ14, Vβ19 and Vβ24) [15]. In the present study, in which flow cytometry was used, increased usage of the Vβ16 chain in the CD4+ T cells of peripheral blood was found in the majority of SLE children. Higher values of other CD4+ Vβ chains were also noticed in SLE children in comparison to healthy ones, but they concerned individual patients. Our results are difficult to compare with previous studies published in adults, not only because of the different methodologies used (flow cytometry versus spectratyping) or to different samples studied (children versus adults, patients at initial diagnosis versus patients under treatment) but also because of the limited number of the studies conducted in that field. Larger scale analysis in populations categorized according to age, to time of the study held (before or after treatment), to sex, to ethnicity and to methodology used could provide further insight on the role of Vβ repertoire not only in autoimmune disease pathogenesis but also in its clinical significance as a prognostic factor in the case of SLE.

Limitations of the present study are the limited number of the patients studied, but this is due to the rarity of lupus in children in the Greek population and to the fact that only patients upon the initial diagnosis were selected in order to avoid alterations in their immunological profile that medication could induce. Another parameter is the fact that evaluation of the Vβ repertoire in each patient is not influenced by the number of the patients investigated. Although genetic evaluation was not performed, all previous published studies in immune mediated diseases have showed that there are no discrepancies between the results from flow cytometric analysis and spectratyping, enhancing the reliability of flow cytometry. Finally, the CD8+ Vβ lymphocyte subpopulations were not analyzed, and therefore it is not known whether there are abnormalities in the repertoire of children with diabetes or lupus or not.

As far as immunophenotyping analysis is concerned, no major differences among the groups were noticed. Regarding the role of the CD5 + CD19+ population in autoimmune process, its expression was quite similar in the three groups studied. This finding is in accordance with other studies in adult SLE patients, but not with studies in T1DM patients, in whom increased expression was observed [20,21].

## Conclusions

This is the first comparative flow cytometric analysis between an organ-specific autoimmune disease, T1DM, and a systemic one, SLE, which underscores the importance of the TCR Vβ repertoire in patients with auto-immune diseases. This study was possible because of the availability of an easily applicable method at a clinical setting, which enabled TCR Vβ repertoire analysis prior to initiation of any treatment that could possibly be a confounding factor. Abnormalities of the CD4+ TCR Vβ repertoire quantitative expression were observed mainly in SLE children. Low usage of certain CD4 + TCR Vβ chains was also far more prominent in lupus patients. Only the CD4 + TCR Vβ4 and CD4 + TCR Vβ16 lymphocytes were significantly increased in T1DM and SLE children, respectively. However, the potential role of these lymphocyte clones in the pathogenesis of autoimmunity remains to be clarified.

## Methods

### Subjects

Samples were collected from a) Fifteen newly diagnosed children with T1DM (mean age ± SD: 9.2 year ± 4.78, 11 males-4 females) during their hospitalization upon diagnosis in the First Department of Pediatrics of the University of Athens in “Aghia Sophia” Children’s Hospital and b) Nine patients with SLE (mean age ± SD: 12.8 years ± 1.76, 3 males-6 females) upon diagnosis and prior to treatment initiation, all of Hellenic origin. Thirty one healthy Greek children (mean age ± SD: 6.58 years ± 3.65, 26 males-5 females) undergoing minor surgery at the First and Second Department of Surgery at “Aghia Sophia” Children’s Hospital were included as controls. Taking into account that several germ line polymorphisms of the TCR Vβ genes have been described in different ethnicities [22,23], this study was restricted to children of Greek origin in order to include an ethnically homogeneous population. A standardized interview was held with parents of all children.

### Ethical approval

The study was approved by the Ethical Committee of the “Aghia Sophia” Children’s Hospital and participants were included in the study only after informed consent was obtained from their parents or guardians.

## Methodology

Blood (4 ml in EDTA tube and 3 ml serum) was taken from all participants for investigations, including full blood count, serum immunoglobulin levels assessment by nephelometry and screening for autoantibodies by indirect immune-fluorescence (ANA, anti-dsDNA, ICA), as well as lymphocyte subpopulation immunophenotyping by flow cytometry.

### Flow cytometry

CD4 + TCR Vβ repertoire was analyzed using three-color flow cytometry with the IOTest Beta Mark TCR Repertoire Kit (Beckmann Coulter, Marseille, France), which consists of fluorochrome conjugated monoclonal antibodies that identify 24 TCR Vβ subfamilies, covering about the 70% of the normal human CD4+ T cells. Since at the time of the study initiation, only three-color flow cytometry was in practice, and in order to assess the results with the same method, no changes in methodology were undertaken. For that reason only the CD4+ TCR Vβ repertoire and not the CD8+ one was assessed. Peripheral blood samples were collected in tubes containing anticoagulant (EDTA) and were stained within 2 hours. 100 μl of blood was incubated for 20 minutes, at room temperature with 20 μl of a mixture of three distinct anti-Vβ monoclonal antibodies and 10 μl of anti-CD4-PC5 monoclonal antibody (MoAb). Erythrocytes were lysed by NH_4_Cl solution. At least 10.000 CD4+ lymphocytes were collected for analysis. CD4+ lymphocytes were gated using forward scatter, side scatter and FL4 fluorescence; whereas Vβ repertoire analysis used two additional fluorescence channels (FL1 and FL2).

Data acquisition was performed initially on an EPICS XL (Beckman Coulter) flow cytometer and, later on, on an FC-500 (Beckman Coulter), instrument. Listmode analysis was performed using the CXP software.

Lymphocyte subpopulations tested were: 1) CD3+ T cells, 2) helper T cells (CD3 + CD4+), 3) cytotoxic T cells (CD3 + CD8+), 4) natural killer cells (CD56+) and 5) B lymphocytes (CD19+). Moreover, the αβ and γδ expression on T cells, the expression of CD69+ and HLA-DR + on T cells as activation markers and the expression of CD5+ on CD19+ B cells were studied.

### Statistical analysis

Statistical analysis among three age groups of the healthy individuals included in the study was performed in order to detect possible skewing among them. Age was transformed to categorical variables, thus constructing three age groups: 18 months-4^11/12^ years (n = 12), 5-9^11/12^ years (n = 10) and ≥ 10 years (n = 9). The reason for this transformation was to reveal a possible difference in Vβ repertoire usage among specific age groups that roughly reflect the stages of child development and immune system maturation. Vβ repertoire expression variables are described as median and interquartile range IQR (75th-25th percentile) due to the small sample size and to their non-normal distribution. For the same reason, non parametric statistics were used; Mann–Whitney U statistic was used for the comparison of the aforementioned variables between two age subgroups and Kruskal-Wallis rank test when comparing the same variables among the three age subgroups of the study. All tests were two-sided at a significance level of p < 0.5. Data were analyzed using STATA™ (Version 9.0, Stata Corporation, College Station, TX 77845, USA).

To assess the possible differences of CD4 + TCR Vβ repertoire expression, pediatric patients with newly diagnosed SLE and T1DM were compared with healthy age-matched controls. Due to the small subgroup sizes of SLE and T1DM (n = 9, n = 15 respectively), median and IQR (75^th^ - 25^th^ percentile) were used to describe the continuous variables. In addition, nonparametric statistical analysis (Mann–Whitney U) was used to test for any significant differences between the subgroups. Due to the multiple comparisons (n = 24), Bonferroni adjustment was performed in order to correct for inflation in type I error, setting the significance level from p < 0.05 to < 0.0021 (=0.05/24). Statistical analysis was also adjusted to a CV of 20%, which could characterize the results of Vβ chains with rare expression. The significant difference that was found between controls and T1DM patients in Vβ4 chain was also present when using Student’s t-test for the comparison. Although t-test is a parametric statistic and less powerful in our case due to the small number of patients, it allowed us to perform new hypothetical comparisons between the aforementioned groups in order to verify that the comparisons would remain significant even after increasing the mean value in the control group by 20% and decreasing the mean value in the T1DM group (for Vβ4 chain) by 20%.

## Abbreviations

TCR: T Cell receptor; T1DM: Type 1 diabetes mellitus; SLE: Systemic Lupus Erythematosus; IQR: Interquantile range; CV: Coefficient of variation.

## Competing interests

We disclose any financial competing interests. There are no non-financial competing interests to declare in relation to this manuscript.

## Authors’ contributions

FT carried out the study (collection of patients and controls, conduction of flow cytometric analysis and autoantibody screening) and has written the manuscript. MK has significantly contributed in the conception and design, the acquisition of data, the analysis and interpretation of data as well as in the drafting the article and in revising it critically for important intellectual content. She has also significantly contributed in the final approval of the version to be published. MT contributed in the analysis and interpretation of the data and revised the manuscript. CM has significantly contributed in the acquisition of data and the analysis and interpretation of data as well as in the drafting the article and revising it critically. More specifically due to his high qualifications in statistical analysis and interpretation he has contributed in the interpretation and presentation of the important findings of the study. EP has personally conducted the measurement of serum immunoglobulin levels and the assessment and screening for autoantibodies and she has contributed to the interpretation of data. GC revised the manuscript critically for important intellectual content. CKG contributed in the interpretation of the collected data and the preparation of the manuscript. Together with MK, she had the main contribution in the drafting of the article and in revising it critically for important intellectual content. She has also significantly contributed in the final approval of the version to be published. All authors read and approved the final manuscript.

## Authors’ information

**FT** is the PhD candidate, who has conducted the study under the supervision of Dr. Maria Kanariou, the Director of the Department of Immunology & Histocompatibility, “Aghia Sophia” Children’s Hospital, Greece and the help of the members of the Department of Immunology & Histocompatibility. She holds master degree in “Molecular Medicine” (supervisor of the two-year training in basic research methodology is Professor Nikolaos Anagnou). She is Pediatrician and is currently Consultant at the 3^rd^ Department of Pediatrics’ Clinics of “Attikon” University Hospital.

**MK** is the Director of the Department of Immunology & Histocompatibility, Specific Center & Referral Center for Primary Immunodeficiencies - Paediatric Immunology, “Aghia Sophia” Children’s Hospital, Greece where all laboratory measurements have been conducted under her supervision.

**MT** is a Medical Biopathologist with over a decade of experience in Flow Cytometry. She is currently responsible for the Flow Cytometry and Cell Culture Laboratories of the Department of Immunology & Histocompatibility, Specific Center & Referral Center for Primary Immunodeficiencies - Paediatric Immunology, “Aghia Sophia” Children’s Hospital.

**CM** is GP, Consultant at Hospital of Kimi, Greece and holds a master degree in biostatistics and PhD diploma in epidemiology. He also has many publications in several studies in which he contributed as a biostatistician.

**EP** is also a staff member of the Department of Immunology & Histocompatibility, “Aghia Sophia” Children’s Hospital, Greece.

**GC** is the Medical Director of the First Department of Pediatrics and the Division of Endocrinology, Diabetes and Metabolism, of the National Kapodistrian University of Athens, “Aghia Sophia” Children’s Hospital, Greece.

**CKG** is the supervisor of Mrs Tzifi’ PhD thesis and the scientific supervisor of the Diabetes Center, where the type 1 diabetes patients are followed. She is Associate Professor for Pediatric Endocrinology and Juvenile Diabetology in the First Department of Pediatrics and the Division of Endocrinology, Diabetes and Metabolism, of the Medical School of the National Kapodistrian University of Athens, “Aghia Sophia” Children’s Hospital, Greece.
